# Formation of Starch–Lipid Complexes during the Deep-Frying Process and Its Effects on Lipid Oxidation

**DOI:** 10.3390/foods11193083

**Published:** 2022-10-05

**Authors:** Xueying Hu, Zhaoyang Li, Fengyan Wang, Hongyan Mu, Liping Guo, Junxia Xiao, Yuanfa Liu, Xiaodan Li

**Affiliations:** 1 College of Food Science and Engineering, Qingdao Agricultural University, Qingdao 266109, China; 2 Qingdao Special Food Research Institute, Qingdao 266109, China; 3 COFCO Nutrition & Health Research Institute, Beijing 102209, China; 4 State Key Laboratory of Food Science and Technology, School of Food Science and Technology, National Engineering Research Center for Functional Food, National Engineering Laboratory for Cereal Fermentation Technology, Collaborative Innovation Center of Food Safety and Quality Control in Jiangsu Province, Jiangnan University, Wuxi 214122, China

**Keywords:** starch–lipid complexes, lipid oxidation, deep-frying process

## Abstract

In the present study, maize starch (MS), potato starch (PS), high-amylose maize starch (HAMS), and wheat starch (WS) were deep-fried in soybean oil that was continuously heated for 40 h under 180 °C. The thermodynamic and pasting properties of deep-fried starch samples were determined. The results suggested that starch–lipid complexes formed with the extension of frying oils’ usage; however, their number was not dependent on the frying oils’ life cycle. Importantly, the results of pasting properties revealed the following strength of intermolecular force in deep-fried starch samples: PS > MS > HAMS > WS. The results of XRD and FTIR analysis confirmed the formation of starch–lipid complexes during the deep-frying process. Furthermore, the results of the in vitro digestibility of deep-fried starch revealed that the formation of starch–lipid complexes inhibited the swelling of starch granules and prevented the entrance of amylase into the interior. Additionally, the results of the oxidation stability of deep-frying oil indicated that the formation of starch–lipid complexes did not alter the trend of lipid oxidation as an effect of the limited number of starch–lipid complexes. These results could have critical implications for the development of healthier deep-fried foods.

## 1. Introduction

Deep-frying is one of the most common cooking methods, during which foodstuff acquires a golden color, crisp texture, and pleasant flavor. However, oxidation, hydrolysis, and polymerization of triglycerides (TAGs) continue to occur during the deep-frying process [1]. In particular, unsaturated fatty acids in glycerides are prone to oxidization under high temperature and moisture levels, producing oxidized glyceride compounds. Moreover, the generated substances participate in the further reaction of oils, and as a result starch gelatinization and protein denaturation in foodstuff occur, which might result in the loss of nutrients in deep-fried foods [2].

Starchy food is one of the most prevalent kinds of deep-fried foods. To assess the health value of deep-fried starchy food, starch–lipid complexes, which have been identified in deep-fried foods, have attracted the attention of researchers. Scientists found starch–lipid complexes in deep-fried foods such as instant noodles and batter [3]. Researchers indicated that the formation of hydrophobic ligands in amylose was due to the transfer of hydrophobic ligands from polar water to the inner spiral structure of starch and the reaction of fatty acids or glycerides in lipids with starch [4]. During the deep-frying process, the thermodynamic properties, crystal structure, and digestion characteristics of starch would be significantly changed [5,6,7,8,9].

Yang et al. illustrated that the increase of deep-frying temperature would lead to an increase of amylose content [5]. They also found that the formation of starch–lipid complexes could increase the change of enthalpy value and reduce the viscosity of the starch from deep-fried foods. According to the previous researchers, the crystal structure of starch–lipid complexes was associated with the types of fatty acids. In detail, the particle size of the starch–lipid complexes became wider and higher with the increase of the unsaturation degree of the fatty acids [6,7,8,9]. Moreover, Wang et al. [10] suggested that shorter-chain fatty acids produced more starch–lipid complexes compared to long-chain fatty acids. The series studies of Chen et al. [11,12,13] have investigated the effects of granule size and pretreatment of starches on the formation of starch–lipid complexes. Their studies found that the oil absorption of starch was negatively associated with its granule size under limited moisture levels. Li et al. [14] studied the influence of different deep-frying time and temperature on the formation of starch–lipid complexes. Their results indicated that the content of starch–lipid complexes increased with the elevation of temperature below 180 °C, while it declined with the increase of temperature above 180 °C. Additionally, the effect of the water content of starches and protein addition on the formation of starch–lipid complexes have also been investigated in previous studies [15,16,17]. Their results suggested that the starch–lipid complexes formed during the deep-frying process were mostly at a moisture content of 40%, and the addition of protein favored the formation of starch–lipid complexes.

Starch–lipid complexes are regarded as a new type of resistant starch [14]. There is sufficient evidence that starch–lipid complexes exhibited low digestibility, resulting from the formation of V-amylose [18,19]. Some researchers have elucidated that starch–lipid complexes reduced the enzyme sensitivity depending on the composition of fatty acids. Specifically, monounsaturated fatty acids decrease starch digestibility to a greater extent than other fatty acids [20]. Moreover, previous studies have suggested that the inhibition of starch expansion and solubility by the starch–lipid complexes could delay the gelatinization of starch and inhibit its sensitivity to enzymes [21]. Chung et al. [22] found that starch digestibility was affected by the crystallinity and molecular structure of amylose and starch–lipid complexes. Although the formation and physicochemical properties of starch–lipid complexes under deep-frying conditions have been discussed widely, fewer studies have elucidated the effect of starch–lipid complexes’ formation on the oxidative stability of deep-frying oils. Thus, the present study may provide important implications for healthier deep-fried food production.

In this paper, maize starch (MS), potato starch (PS), high-amylose maize starch (HAMS), and wheat starch (WS) were used for the preparation of nuggets with distilled water, and they were then deep-fried under real circumstances. Specifically, the starch samples were fried in soybean oil for 8 min under 180 °C, and the frying oil was fried continuously for 40 h. Starch–lipid complexes formed during the deep-frying process were detected. The thermodynamic properties and crystal structure of deep-fried substances were analyzed to determine the formation of starch–lipid complexes. The in vitro digestibility of deep-fried substances was investigated to illustrate the effect of starch types and deep-frying oils’ life cycle on the properties starch–lipid complexes. Furthermore, the effect of starch–lipid complexes’ formation on the oxidative stability of deep-frying oils was examined.

## 2. Materials and Methods

### 2.1. Materials

WS was purchased from Qufeng Food Tech. Co., Ltd. (Weifang, China); HAMS was purchased from Ruitai Gaozhi Biotechnology Co., Ltd. (Wuhan, China); MS, PS, potassium bromide, α-amylase, and anhydrous sodium acetate were all purchased from Macklin Biochemical Co., Ltd. (Shanghai, China); soybean oil was purchased from Wilmar International Limited (Qingdao, China); DNS chromogenic agent was provided by Solarbio Science & Technology Co., Ltd. (Beijing, China); α-Amyloglucosidase was purchased from BOSF Co., Ltd. (Hefei, China).

### 2.2. Preparation of Deep-Fried Starch Samples

Starch and distilled water were mixed according to a certain mass ratio (6:5). The evenly mixed mixture of starch and water was then divided into a 2.5 cm × 2.5 cm × 1 cm mold to set. The mixture (30 g) was carefully removed and fried for 8 min in soybean oil (3000 g) under a temperature of 180 °C. The size of the fryer was 30 cm × 15 cm × 14 cm. The deep-fried oil was then kept at 180 °C for 12 min before another group of mixtures was processed. The soybean oil was kept at 180 °C for 8 h per day, and the deep-frying process lasted up to 5 d (40 h).

The deep-fried starch samples were ground and defatted with petroleum. The samples were then sifted through 50 meshes.

### 2.3. Determination of Amylose Content

Amylose content was determined based on the method of Zhu et al. [23], with some modifications. Starch samples (100 mg) were dispersed in 10 mL of KOH solution (1 mol/mL) and heated in a water bath (85 ± 1 °C) until all the starch was dissolved. Subsequently, the starch suspension was diluted and the pH was adjusted to 3.0 with HCL (0.1 mol/mL). Iodine reagent (0.2%) was then added, and the reaction took place in a brown volumetric flask.

### 2.4. Analysis of Thermodynamic Properties

The thermodynamic properties of deep-fried starch samples were determined via differential scanning calorimetry (DSC), based on the method of Yang et al. [5], by making some modifications as follows: Deep-fried starch samples (3 mg) were accurately weighed in an alumina crucible with distilled water (9 μL). The samples and water were evenly mixed, and the mixture was equilibrated for 6–8 h at room temperature. The temperature range was set as 50~150 °C with a heating rate of 10 °C/min, and parallel experiments were performed.

### 2.5. Analysis of Pasting Properties

The viscosity of deep-fried starch samples was measured using a rapid viscosity analyzer (RVA). The specific operations were as follows: Deep-fried starch samples (3 g) were placed in a test canister. Distilled water (25 g) was then added to the canister to obtain a starch suspension. Before being loaded, the suspension was stirred several times with a plastic paddle to mix evenly. The test procedure was set as follows: The suspension was heated at 50 °C for 1 min, and it was then heated to 95 °C with a rate of 12.8 °C/min. Finally, the samples were cooled to 50 °C with a cooling rate of 12.8 °C/min and kept at 50 °C for 2 min.

### 2.6. X-ray Diffraction (XRD) Analysis

The wide-angle X-ray diffraction was performed, referring to the method of Chen et al. [24], with some modifications. Specifically, the deep-fried starch samples (50 mg) were tested at 40 kV acceleration voltage and 30 mA current to characterize the crystallinity of starch samples. The diffraction range was between 4° and 40°, and the step size was 0.05°. JADE 6 was used to calculate the relative crystallinity of the samples.

### 2.7. Fourier Transform Infrared (FTIR) Analysis

The method of FTIR was modified by Wang et al. [15]. The fine-ground deep-fried starch samples (1.5 mg) were accurately weighed and mixed with solid potassium bromide (145 mg). The scanning was carried out in the range of 400~4000 cm^−1^, with a resolution of 4 cm^−1^ for 64 scans.

### 2.8. In Vitro Digestibility

The content of rapidly digestible starch (RDS), slowly digestible starch (SDS), and resistant starch (RS) in the deep-fried starch samples was analyzed according to the method described by Kang et al. [16]. Specifically, fine-ground deep-fried starch samples (100 mg, dry base) were accurately weighed into a centrifuge tube with sodium acetate buffer (4 mL, pH 5.2, 0.1 mol/L), and the mixture was mixed well via a vortex. The centrifuge tube was bathed in boiling water for 30 min. After the mixture was cooled to room temperature, supernatant (0.2 mL) was removed and added into a 25 mL stopper tube. Distilled water (1.8 mL) and DNS reagent (2 mL) were then added to the stopper tube. Subsequently, the tube was placed in boiling water for 5 min, and distilled water was added up to 25 mL. Finally, OD_540nm_ of the solutions was recorded, and the glucose content was calculated (G_0_).

α-Amylase (1.0 g) and amyloglucosidase (20 μL) were added to the centrifuge tubes with the deep-fried starch samples and sodium acetate buffer, as mentioned above. The tubes were then shaken well and incubated under 37 °C. Enzymolysis solution (0.2 mL) was mixed with anhydrous ethanol (4.8 mL) at 20 min (G_20_) and 120 min (G_120_), respectively. The mixture was then centrifugated at 3000 r/min for 10 min, and the supernatant (0.5 mL) was added to a test tube with distilled water (1.5 mL). G_0_, G_20_, and G_120_ are called RDS, SDS, and RS, respectively.

RDS/% = 0.9 × (G_20_ − G_0_)/total starch mass × 100

SDS/% = 0.9 × (G_120_ − G_20_)/total starch mass × 100

RS/% = 100 − RDS% − SDS%

G_0_: glucose content at 0 min of enzymatic hydrolysis;

G_20_: glucose content at 20 min of enzymatic hydrolysis;

G_120_: glucose content at 120 min of enzymatic hydrolysis;

0.9: conversion coefficient.

### 2.9. Determination of Oxidative Stability

The peroxide value (POV) and *p*-anisidine value (*p*-AV) of deep-frying oil were determined according to the official methods of the American Oil Chemists’ Society Cd 8b-90 and Cd 18–90, respectively. The total oxidation value (TOTOX) was calculated based on the equation:

TOTOX = 2POV+ *p*-AV

### 2.10. Data Analysis

All analyses were performed in triplicate, and the results are presented as mean ± standard deviation (Mean ± SD). The statistical significance of the data was analyzed by one-way ANOVA and Tukey’s tests (*p* < 0.05). The SPSS package was used for all statistical analysis. The data graphs were plotted using Origin 2017.

## 3. Results and Discussion

### 3.1. Determination of Amylose Content

As can be observed in Figure S1, the amylose content of native HAMS (33.86%) was significantly higher than that of the other types of native starch samples (21%~25%), a finding that is consistent with that of a previous study [25]. It has been reported that the amylose content was positively associated with starch granule size [13]. Generally, MS granules have diameters in the range of 2~30 μm, and the diameters of potato and wheat starch granules were 10~110 μm and 10~35 μm, respectively [26,27]. During the deep-frying process, starch granules expanded and their integrity was destroyed [26].

As shown in Figure S1a, no significant difference was found in the amylose content of deep-fried MS during the deep-frying process, nor in that of native MS. The different results are illustrated in Figure S1d, where WS was studied. The amylose content of deep-fried WS at 40 h (28.99%) was significantly higher than that of native WS. However, there was no significant difference among deep-fried WS samples obtained at other frying times (8 h, 16 h, 24 h, and 32 h) and native WS. Interestingly, the amylose content of deep-fried PS increased significantly in comparison with native PS, and it fluctuated with the extension of the deep-frying procedure (Figure S1b). Previous studies by Chen et al. [13,26] have elucidated that, compared to the larger starch granules, the smaller ones were more stable to thermal treatment. Therefore, the greater quantity of amylose in PS was leached during the deep-frying process in comparison with the starches with smaller diameters, specifically the maize and wheat starch in the present study. Notably, the amylose content of deep-fried HAMS was significantly decreased compared to native HAMS (Figure S1c). Amylose is known for its tendency to form single helices, which tend to accommodate hydrophobic compounds, such as lipid molecules. Thus, starch–lipid complexes were formed more easily under heat treatment as a result of a high content of amylose in HAMS. On the other hand, the presence of starch–lipid complexes limited the expansion of starch granules [26], and a smaller amount of amylose would be leached. Herein, the amylose content of deep-fried HAMS was significantly lower than that of native HAMS. Moreover, it was obvious that the frying oils’ life cycle (8~40 h) did not alter the amylose content of the MS, HAMS, and WS samples assessed in the present study, and the amylose content of deep-fried PS fluctuated during the deep-frying process.

### 3.2. Analysis of Thermodynamic Properties

The effect of the deep-frying process on the thermodynamic properties of starch samples is shown in Table 1. Previous studies reported that the characteristic thermal transition temperature of native starch ranged from 60 °C to 80 °C [5,11]. As indicated in Table 1, the starch gelatinization peak (*T*_P_ = 68.82 °C) of MS disappeared, and new ones were found between 122.78 °C and 141.69 °C during the deep-frying process, indicating the gelatinization of starch and the formation of starch–lipid complexes [5]. The melting enthalpy (Δ*H*) of deep-fried MS samples was significantly lower than that of native MS (9.95 J), suggesting the formation of starch–lipid complexes during the deep-frying process [5,11,12,28]. It has been reported that the Δ*H* of amylose-lipid complexes is an indicator of the amount of complex and the degree of crystallinity in the starch, which is mostly related to the amount of complex [10,28]. Notably, the Δ*H* of deep-fried MS samples at 32 h (6.61 J) was the highest among the samples where the oil had been heated from 8 h to 40 h. A previous study found that the structure of starch–lipid complexes with palmitic acid (C16:0), myristic acid (C14:0), and lauric acid (C12:0) was more ordered than that of complexes with stearic acid (C18:0), oleic acid (C18:1), and linoleic acid (C18:2) [29]. Moreover, another study found that the amount of starch–lipid complex declined with the increase of fatty acids’ chain length [10]. In addition, greater and more dispersed starch granules were formed with the increase of fatty acids’ unsaturation [30]. Soybean oil, where linoleic acid (C18:2) was the predominant fatty acid composition, was applied in the present study. The fatty acid composition altered with the usage time of the frying oils. Specifically, the oxidation of linoleic acid (C18:2) might lead to the formation of lauric acid (C12:0) during the deep-frying process [31]. Thus, more highly-ordered starch–lipid complexes formed with the extension of frying oils’ usage.

The gelatinization peak (63.31 °C) of native PS disappeared and new ones were found between 131.90 °C and 145.10 °C in deep-fried PS samples, indicating the melting of starch–lipid complexes. The Δ*H* of deep-fried PS samples was significantly lower than that of untreated PS (16.60 J), and the Δ*H* of deep-fried PS samples at 40 h (0.58 J) had the lowest value. The results suggested the formation of starch–lipid complexes during the deep-frying process. A similar trend was observed in HAMS and WS, where the gelatinization peak of native starch disappeared and new ones were found in deep-fried starch samples, indicating the formation of starch–lipid complexes during the deep-frying process.

### 3.3. Analysis of Pasting Properties

The effect of the deep-frying procedure on the pasting properties of the four types of starch is presented in Table 2. The peak viscosity of MS decreased significantly from 2926 cP (native) to 2328 cP during the deep-frying process, while no significant difference was observed among the deep-fried MS samples from 16 h to 40 h. The results suggested that the swelling of starch granules was inhibited because of the formation of starch–lipid complexes, and this was consistent with previous studies [4,16]. In addition, Chen et al. suggested that the formation of smaller molecular fractions resulting from the degradation of starch granules during the deep-frying process also contributed to the decrease of peak viscosity [32]. Although it has been reported that the barrier effect of the oil on starch particles was the main reason for viscosity reduction [33], in the present study, the deep-fried starch samples were defatted before being analyzed. A similar trend was observed in the breakdown viscosity of MS, where the viscosity of native MS decreased by over 50% during the deep-frying process, indicating a strong intermolecular force in deep-fried starch samples [5]. On the other hand, the final and setback viscosity of MS significantly increased with the deep-frying treatment, and the results were in line with previous studies [4,10,16,29]. The melt and re-solidification of fatty acids within starch–lipid complexes was attributed to the increase of final and setback viscosity [4,10].

As in MS, a similar trend was observed in the viscosity for the other types of starch investigated in the present study. Notably, the peak viscosity of PS and HAMS decreased the most (more than 50%), and that of MS declined the least. Interestingly, the breakdown viscosity of PS decreased by more than 80% during the deep-frying process. However, there was no significant difference between the breakdown viscosity of native WS and that of deep-fried ones, indicating that the intermolecular force of deep-fried WS was weaker than the others. A significant increase in the final and setback viscosity of PS during the deep-frying process is presented in Table 2, which was the same as MS. However, the final viscosity of HAMS and WS decreased significantly with the treatment. A previous study showed that starch granules immersed in oil tended to polymerize and were then deposited on the surface of starch particles, leading to a decrease of amylose leaching [5,32]. It is worth noting that the gelatinization of HAMS occurs when the temperature reaches 120 °C or above [34]. Thus, the pasting properties of HAMS were not distinct under the temperature of 100 °C. Although no significant difference was observed between the setback viscosity of native WS and that of deep-fried WS at 8 h and 40 h, the setback viscosity of deep-fried WS at 16 h, 24 h, and 32 h was significantly lower than that of native WS. Overall, the pasting properties of deep-fried starches relied more on the characteristics of starch (amylose content, moisture content, etc.) in comparison with the life cycle of frying oil.

### 3.4. Analysis of XRD

**Figure 1:**
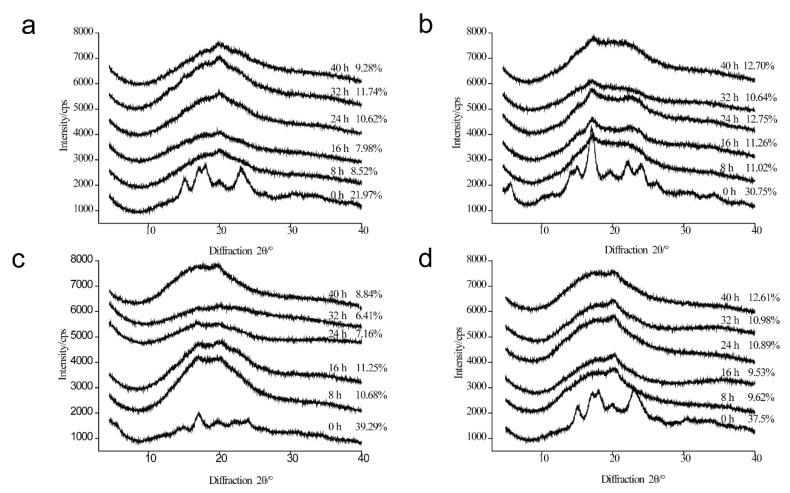
X-ray diffraction (XRD) patterns of native maize starch, potato starch, high-amylose maize starch, and wheat starch (0 h), as well as those during the deep-frying process (8 h, 16 h, 24 h, 32 h, 40 h). (**a**) Maize starch; (**b**) Potato starch; (**c**) High-amylose maize starch; (**d**) Wheat starch.

The diffraction patterns and relative crystallinity of four types of starch sample during the deep-frying process are displayed in Figure 1. As shown in Figure 1a, the characteristic A-type diffraction peaks at 15°, 17 °, and 24° (2θ) of MS disappeared during the deep-frying process [26], and a new peak at 20° is shown, indicating the formation of starch–lipid complexes [9]. The results are in agree with previous studies [11,35]. The relative crystallinity of the deep-fried MS samples increased with the extension of deep-frying time, and the maximum relative crystallinity (11.74%) was detected at 32 h’s usage of deep-frying oil. The results were consistent with DSC.

As for PS, the diffraction peak at 15°, 20°, 22°, and 23° (B-type starch) disappeared, while the diffraction peak at 17° was presented either in native PS or deep-fried PS samples (Figure 1b). Interestingly, a weak diffraction peak at 22° was observed in deep-fried PS, indicating the aggregation of fatty acids [29,35]. In comparison with A-type starch, B-type starch is less densely packed [26]. Overall, the results suggested that the B-type crystalline structures of PS were destroyed. The relative crystallinity of deep-fried PS in Figure 1b was a result of the synergistic effect of higher temperature and increased amylose content instead of an indicator of the amounts of starch–lipid complexes formed during the deep-frying process.

The diffraction peaks of HAMS, which contains a high level of B-type starch, disappeared, except for the peak at 17° during the deep-frying process (Figure 1c). Additionally, a new diffraction peak at 21° appeared during the deep-frying process, suggesting the formation of V-type complexes in deep-fried HAMS. Notably, there was no correlation between the relative crystallinity of deep-fried HAMS and the life cycle of frying oil. Specifically, the relative crystallinity of deep-fried HAMS obtained at 16 h remained at the highest level (11.25%) during the deep-frying process, while the relative crystallinity of deep-fried HAMS decreased sharply to 6.41% (32 h) with the extension of frying oils’ life cycle. Subsequently, it increased gradually to 8.84% (40 h). As illustrated in Figure 1d, the characteristic A-type diffraction peaks of wheat starch disappeared during the deep-frying process, and a new peak at 20° was shown in the deep-fried wheat starch samples. The results indicated the formation of starch–lipid complexes in wheat starch during the deep-frying process. Notably, the relative crystallinity of deep-fried wheat starch increased with the extension of frying oils’ usage time, indicating that the amount of starch–lipid complexes increased during the deep-frying process.

### 3.5. Analysis of FTIR

**Figure 2:**
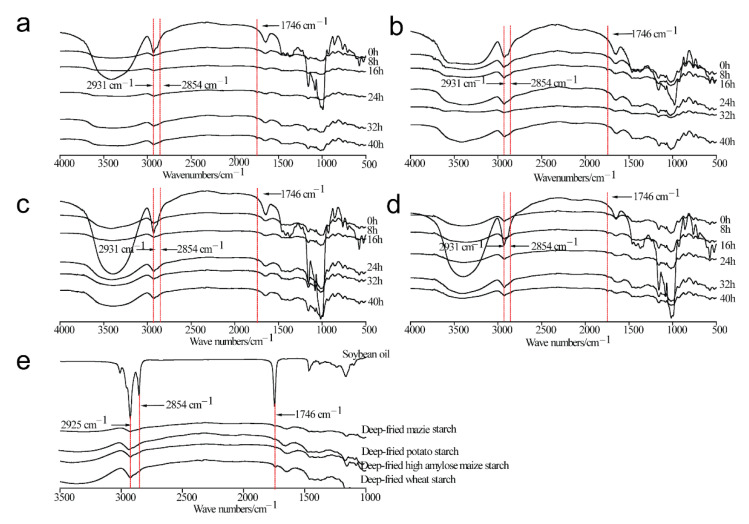
Fourier transform infrared (FTIR) spectra of native maize starch, potato starch, high-amylose maize starch, and wheat starch (0 h), as well as those during the deep-frying process (8 h, 16 h, 24 h, 32 h, 40 h). (**a**) Maize starch; (**b**) Potato starch; (**c**) High-amylose maize starch; (**d**) Wheat starch; (**e**) Soybean oil and deep-fried starch samples at 8 h.

The FTIR spectra of four types of deep-fried starch samples are displayed in Figure 2a–d. Obviously, the absorption band at 3425 cm^−1^ (-OH) of all the starch samples investigated in the present study was significantly weakened during the deep-frying process, suggesting the ordered structure of starch granules was destroyed and the intramolecular or intermolecular hydrogen bonds were weakened. In addition, the intensity of -CH_2_ and -CH absorption bands (2931 cm^−1^) was weakened during the deep-frying process, as was that of the C-O-H absorption peak (1080 cm^−1^), indicating that the crystalline structure of starch particles was destroyed. The results were in line with previous studies [15,16,36]. As shown in Figure 2e, absorption peaks of the -C-H stretching vibration of soybean oil were observed at 2925 cm^−1^ and 2854 cm^−1^ [15,16,36]. In addition, the absorption band at 1746 cm^−1^ was assigned to -C=O stretching vibration [36]. In comparison with untreated starch samples, the absorption peaks at 2854 cm^−1^ and 1746 cm^−1^ were observed in four types of deep-fried starch samples, where the bands indicated the vibrations of -C-H and -C=O in soybean oil, suggesting the presence of lipid in deep-fried starch samples. Therefore, the formation of starch–lipid complexes during the deep-frying process was confirmed. Although lipid was observed in native starch samples (Table S1), the lipid content was much lower than the amylose content. Thus, the lipid in starch–lipid complexes was from the deep-frying oil.

### 3.6. Analysis of In Vitro Digestibility

**Figure 3:**
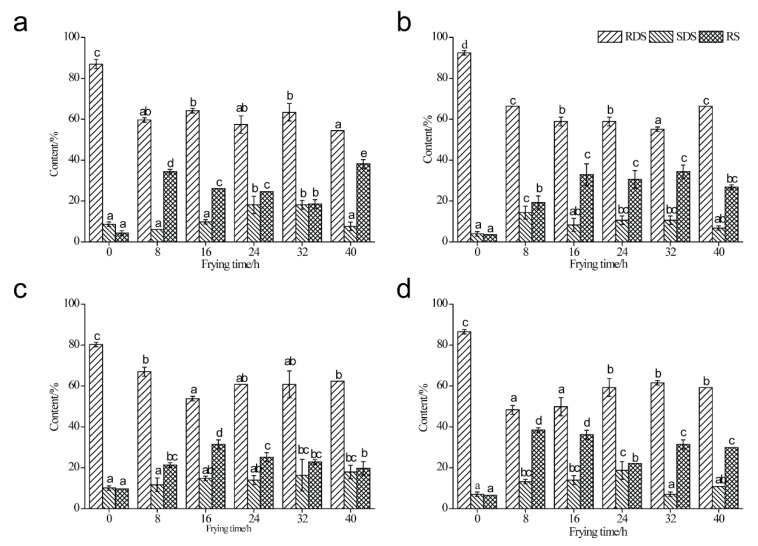
In vitro digestibility of native maize starch, potato starch, high-amylose maize starch, and wheat starch (0 h), as well as those during the deep-frying process (8 h, 16 h, 24 h, 32 h, 40 h). (**a**) Maize starch; (**b**) Potato starch; (**c**) High-amylose maize starch; (**d**) Wheat starch.

The contents of RDS, SDS, and RS of four types of starch samples are shown in Figure 3. Obviously, RS content increased in deep-fried starch samples in comparison with native starch samples, which was in line with the results of previous studies [10,17,35]. As indicated in Figure 3a, the content of RS in MS elevated significantly from 4.36% (untreated) to 38.12% (40 h) during the deep-frying process. The formation of starch–lipid complexes inhibited the swelling of starch granules and prevented the entrance of amylase into the interior of starch particles [4,10,25,36,37]. In addition, the dispersibility of starch granules was restricted and the steric hindrance was enhanced due to the presence of complexes, leading to a decreased digestibility [16,36]. The RS content of deep-fried MS samples increased to 34.34% when the deep-frying oil had been used for 8 h. Subsequently, the content of RS declined significantly to 18.50% (32 h). The RS content in PS increased dramatically from 3.57% (untreated) to 19.25% and 32.84% when the deep-frying oil has been used for 8 h and 16 h, respectively (Figure 3b). Notably, the RS content from 16 h to 40 h fluctuated, and there was no significant difference. The results suggest that starch–lipid complexes were formed in deep-fried PS. As shown in Figure 3c, the RS content of native HAMS was the highest (9.64%) among the starch samples investigated in the present study, and it increased with the extension of the deep-frying process. The highest RS content (31.45%) was recorded under the 16 h’s usage of deep-frying oil, and after that the RS content of deep-fried starch samples decreased gradually to 19.77% (40 h). Interestingly, a dramatical increase of RS content was observed in deep-fried WS samples compared to that of untreated WS (Figure 3d). Specifically, the RS contents of deep-fried WS samples at 8 h (38.46%) and 16 h (36.13%) of deep-frying were significantly higher than those of 24 h, 32 h, and 40 h, where the RS content of deep-fried WS samples at 24 h was the lowest (22.10%).

As shown in Figure 3, the RDS contents of four types of starch samples studied in the present study declined during the deep-frying process compared to untreated starch samples. Interestingly, the RDS contents of deep-fried starch samples fluctuated during the deep-frying process, and frying oils’ life cycle had no effect on the variation of RDS content as to the four types of starch sample. To compare the content of SDS and RS among different types of deep-fried starch samples, the data at 8 h’s usage of deep-frying oil were analyzed. Obviously, SDS and RS content of deep-fried WS samples was the highest (51.70%), and this was followed by those of deep-fried MS (40.38%), PS (33.59%), and HAMS (33.01%). In addition, the increment of SDS and RS content between native and deep-fried HAMS (8 h) was the least, and the highest increment of SDS and RS content between native and deep-fried starch (8 h) was observed in the WS sample. Previous studies have reported that starch source and amylose content were highly associated with the digestibility of starch–lipid complexes [4,26]. In the present study, WS and MS contain high levels of A-type crystalline structures, where the double helixes are densely packed together. On the other hand, PS and HAMS contained high levels of B-type crystalline with less densely packed structures. Thus, the SDS and RS content of deep-fried wheat and maize starch was dominantly higher than that of deep-fried potato and high-amylose maize starch because of the different extents of steric hindrance. Moreover, the amylose content of native HAMS was higher than the other three types of starch (Figure S1), and this resulted in the least increment of SDS and RS content between native and deep-fried HAMS (8 h).

### 3.7. Analysis of Oxidative Stability of Deep-Frying Oil

POV, *p*-AV, and TOTOX values are shown in Table 3. The POV of deep-frying oil with MS increased significantly from 3.63 meq/kg (0 h) to 6.62 meq/kg (24 h) at the first 24 h during the deep-frying process. Then, a gradual decrease of POV was seen from 24 h to 40 h (4.64 meq/kg). As is known, hydroperoxide is unstable, and it is easy to disintegrate and used to further form secondary oxidation products (aldehydes and ketones). Thus, the decrease of POV was attributed to the fact that the fission rate of hydroperoxides exceeded the formation rate. In PS, the POV of deep-frying oil also increased by 33.76% after being used for 40 h. A dramatical increase of POV in the deep-fried HAMS system was observed at 16 h, where the value was more than four times as much as that at 0 h. Subsequently, the POV of deep-fried oil declined to 7.55 meq/kg at the end of the deep-frying process. A similar alteration of POV was observed in the deep-fried WS system, where the value of deep-frying oil increased to 15.12 meq/kg at 16 h. A significant decrease was then observed from 16 h to 40 h. Overall, the alteration of POV of deep-frying oil was time-dependent, and this was consistent with previous studies [38].

Interestingly, the formation and destruction rate of hydroperoxides was different in various types of starch. The highest accumulation of hydroperoxides was observed in the deep-fried WS and HAMS system, indicating a higher lipid oxidation rate, and this was confirmed by the *p*-AV of the deep-frying oils, which was a crucial indicator of secondary oxidation product formation. Meanwhile, the lipid oxidation rate of deep-frying oil with MS and PS was much lower than that with WS and HAMS. As indicated in the present study, the intermolecular force of deep-fried PS and WS was the strongest and weakest, respectively. Overall, the strength of intermolecular force followed the following course: deep-fried PS > deep-fried MS > deep-fried HAMS > deep-fried WS. The results indicated that starch with a strong interaction could maintain the integrity of the swollen granules [39] and bound the water more tightly. Thus, the lipids in the deep-fried WS system were supposed to be exposed to more moisture, and the oxidative stability of deep-frying oil decreased. On the other hand, the oxidative stability of lipids was not altered because of the formation of starch–lipid complexes. Although the helix structure of amylose trapped fatty acids and limited further oxidation, the amount of starch samples used in this study was much less than that of deep-frying oils. Therefore, the formation of starch–lipid complexes could not change the trend of lipid oxidation during the deep-frying process.

## 4. Conclusions

This study investigated the effects of frying oils’ usage time on the formation of starch–lipid complexes in four types of starch, namely MS, PS, HAMS, and WS, as well as the effects of starch–lipid complexes on lipid oxidation. The thermodynamic properties, XRD, and FTIR analysis of deep-fried starch samples suggested that starch–lipid complexes formed during the deep-frying process. In addition, starch–lipid complexes formed with the extension of frying oils’ usage, while no correlation was observed between the amounts of starch–lipid complexes and frying oils’ life cycle. Furthermore, it was revealed that the interaction between starch and lipid relies more on the crystalline structure of native starch in comparison with the usage life of frying oil. Interestingly, the results of the pasting properties of deep-fried starch samples revealed that the strength of intermolecular force followed the following course: deep-fried PS > deep-fried MS > deep-fried HAMS > deep-fried WS. In vitro digestibility analysis showed a dramatical increase of RS content in deep-fried starch samples. However, the results of the present study suggest that the formation of starch–lipid complexes did not alter the trend of lipid oxidation during the deep-frying process, and this fact was attributed to the limited amounts of starch–lipid complexes.

## Figures and Tables

**Table 1:** Thermodynamic properties of starch during the deep-frying process.

Deep-Fried Times/h	To/°C	T_P_/°C	T_C_/°C	Δ*H*/J
Maize starch				
0	64.97 ± 2.04 ^a^	68.82 ± 0.74 ^a^	74.08 ± 0.58 ^a^	9.95 ± 0.08 ^e^
8	138.67 ± 0.97 ^e^	141.69 ± 1.39 ^e^	143.63 ± 1.07 ^e^	3.19 ± 0.04 ^c^
16	131.75 ± 0.47 ^d^	136.15 ± 0.76 ^d^	137.04 ± 0.16 ^d^	1.51 ± 0.07 ^b^
24	139.04 ± 1.43 ^e^	140.96 ± 0.86 ^e^	142.86 ± 1.17 ^e^	1.42 ± 0.02 ^b^
32	124.12 ± 0.69 ^c^	132.47 ± 1.24 ^c^	134.75 ± 0.32 ^c^	6.61 ± 0.16 ^d^
40	119.10 ± 0.43 ^b^	122.78 ± 1.05 ^b^	126.01 ± 0.97 ^b^	0.35 ± 0.01 ^a^
Potato starch				
0	58.74 ± 0.25 ^a^	63.31 ± 0.53 ^a^	69.18 ± 1.61 ^a^	16.60 ± 0.28 ^d^
8	125.43 ± 0.80 ^b^	132.99 ± 1.20 ^c^	136.14 ± 1.08 ^b^	5.33 ± 0.07 ^c^
16	134.70 ± 0.29 ^e^	139.24 ± 0.86 ^d^	143.58 ± 0.64 ^d^	5.67 ± 0.04 ^c^
24	132.12 ± 1.09 ^d^	134.77 ± 0.04 ^c^	140.89 ± 1.14 ^c^	0.84 ± 0.13 ^a^
32	129.05 ± 0.63 ^c^	131.90 ± 0.75 ^b^	134.16 ± 0.62 ^b^	2.86 ± 0.16 ^b^
40	142.56 ± 0.22 ^f^	145.10 ± 0.61 ^e^	147.05 ± 0.15 ^e^	0.58 ± 0.03 ^a^
High-amylose maize starch				
0	69.86 ± 1.03 ^a^	72.88 ± 1.56 ^a^	81.79 ± 2.43 ^a^	5.67 ± 0.29 ^c^
8	124.04 ± 0.39 ^e^	128.11 ± 0.88 ^d^	131.07 ± 0.96 ^b^	1.37 ± 0.04 ^a^
16	106.08 ± 0.18 ^b^	123.88 ± 0.41 ^b^	137.81 ± 0.39 ^b^	4.80 ± 0.21 ^b^
24	106.84 ± 0.50 ^b^	126.21 ± 1.17 ^c^	136.16 ± 0.45 ^b^	4.38 ± 0.13 ^b^
32	119.70 ± 0.45 ^d^	126.93 ± 0.62 ^cd^	131.51 ± 0.47 ^b^	4.80 ± 0.18 ^b^
40	117.36 ± 1.87 ^c^	133.55 ± 1.76 ^e^	147.07 ± 1.43 ^c^	1.40 ± 0.11 ^a^
Wheat starch				
0	55.94 ± 0.48 ^a^	61.47 ± 0.53 ^a^	65.90 ± 0.71 ^a^	7.53 ± 0.08 ^d^
8	140.99 ± 0.57 ^d^	145.13 ± 0.77 ^d^	145.79 ± 2.13 ^d^	1.20 ± 0.14 ^b^
16	127.14 ± 0.92 ^b^	127.38 ± 1.86 ^b^	131.09 ± 1.02 ^b^	0.85 ± 0.06 ^b^
24	127.16 ± 0.32 ^c^	136.42 ± 1.12 ^c^	140.09 ± 0.14 ^c^	6.25 ± 0.30 ^c^
32	144.47 ± 0.68 ^d^	144.83 ± 0.52 ^d^	146.47 ± 1.58 ^d^	0.35 ± 0.02 ^a^
40	126.26 ± 0.43 ^b^	129.87 ± 1.07 b	132.96 ± 0.47 b	1.08 ± 0.06 ^b^

Note: Data with different letters in the same column were significantly different (*p* < 0.05) by Tukey’s test. Statistical analysis was only performed among the samples obtained from the same type of starch.

**Table 2:** Pasting properties of starch during the deep-frying process.

Deep-Fried Times/h	Viscosity/cp					Peak Time/s
	Peak	Trough	Breakdown	Final	Setback	
Maize starch						
0	2926 ± 18.38 ^c^	1788 ± 119.50 ^a^	1139 ± 137.89 ^b^	2863 ± 176.07 ^a^	1075 ± 295.57 ^a^	5.20 ± 0.00 ^a^
8	2568 ± 83.44 ^b^	2050 ± 68.59 ^b^	519 ± 14.85 ^a^	3830 ± 101.12 ^c^	1780 ± 169.71 ^c^	5.77 ± 0.05 ^b^
16	2328 ± 97.58 ^a^	1968 ± 7.07 ^b^	360 ± 104.65 ^a^	3265 ± 30.41 ^b^	1297 ± 37.48 ^ab^	6.00 ± 0.00 ^d^
24	2464 ± 60.81 ^ab^	1998 ± 11.31 ^b^	466 ± 49.50 ^a^	3501 ± 120.21 ^b^	1503 ± 108.89 ^bc^	5.91 ± 0.06 ^c^
32	2336 ± 36.77 ^a^	1944 ± 57.28 ^b^	393 ± 20.51 ^a^	3312 ± 74.95 ^b^	1369 ± 17.68 ^ab^	5.89 ± 0.02 ^c^
40	2427 ± 39.60 ^ab^	2041 ± 3.54 ^b^	387 ± 43.13 ^a^	3472 ± 28.28 ^b^	1432 ± 24.75 ^abc^	5.86 ± 0.01 ^c^
Potato starch						
0	5204 ± 7.07 ^c^	1945 ± 60.10 ^a^	3260 ± 53.03 ^c^	2430 ± 43.84 ^a^	486 ± 103.94 ^a^	3.60 ± 0.00 ^a^
8	2687 ± 24.75 ^b^	2468 ± 9.19 ^d^	219 ± 15.56 ^a^	3492 ± 17.68 ^c^	1024 ± 26.87 ^cd^	5.80 ± 0.00 ^c^
16	2590 ± 22.63 ^ab^	2101 ± 4.24 ^b^	489 ± 26.87 ^b^	2847 ± 73.54 ^b^	746 ± 77.78 ^b^	5.14 ± 0.09 ^b^
24	2551 ± 36.06 ^a^	2433 ± 36.77 ^d^	118 ± 72.83 ^a^	3410 ± 20.51 ^c^	977 ± 57.28 ^c^	5.92 ± 0.02 ^c^
32	2557 ± 52.33 ^a^	2330 ± 51.62 ^c^	228 ± 103.94 ^a^	3506 ± 12.73 ^c^	1177 ± 38.89 ^d^	6.07 ± 0.00 ^c^
40	2540 ± 66.47 ^a^	2334 ± 19.09 ^c^	207 ± 47.38 ^a^	3417 ± 33.23 ^c^	1083 ± 14.14 ^cd^	5.27 ± 0.00 ^b^
High-amylose maize starch						
0	269 ± 12.73 ^b^	184 ± 35.36 ^b^	85 ± 22.63 ^b^	270 ± 20.51 ^b^	86 ± 14.85 ^b^	7.00 ± 0.00 ^b^
8	79 ± 5.66 ^a^	71 ± 4.95 ^a^	9 ± 0.71 ^a^	71 ± 5.66 ^a^	0.50 ± 0.71 ^a^	6.80 ± 0.00 ^a^
16	84 ± 13.44 ^a^	73 ± 11.31 ^a^	11 ± 2.12 ^a^	76 ± 11.31 ^a^	3.00 ± 0.00 ^a^	6.97 ± 0.05 ^a^
24	79 ± 2.83 ^a^	70 ± 1.41 ^a^	9 ± 1.41 ^a^	71 ± 0.71 ^a^	0.5 ± 0.71 ^a^	6.87 ± 0.09 ^a^
32	72 ± 1.41 ^a^	66 ± 2.83 ^a^	6 ± 1.41 ^a^	67 ± 0.71 ^a^	0.5 ± 2.12 ^a^	6.97 ± 0.05 ^a^
40	74 ± 3.54 ^a^	66 ± 5.66 ^a^	8 ± 2.12 ^a^	66 ± 4.24 ^a^	0.00 ± 1.41 ^a^	6.73 ± 0.00 ^a^
Wheat starch						
0	3093 ± 7.07 ^d^	2377 ± 14.14 ^d^	716 ± 7.07 ^a^	3655 ± 64.35 ^d^	1278 ± 50.20 ^d^	5.00 ± 0.00 ^a^
8	1997 ± 33.94 ^ab^	1168 ± 11.31 ^a^	829 ± 45.25 ^ab^	2417 ± 21.92 ^b^	1249 ± 10.61 ^cd^	5.53 ± 0.00 ^d^
16	1997 ± 57.28 ^ab^	1178 ± 22.63 ^a^	819 ± 34.65 ^ab^	2350 ± 52.33 ^ab^	1172 ± 29.70 ^bc^	5.47 ± 0.00 ^cd^
24	2136 ± 59.40 ^a^	1144 ± 28.99 ^a^	993 ± 88.39 ^b^	2273 ± 7.07 ^a^	1130 ± 21.92 ^b^	5.39 ± 0.02 ^c^
32	1936 ± 41.01 ^a^	1253 ± 38.18 ^b^	683 ± 79.20 ^a^	2254 ± 70.71 ^a^	1001 ± 32.53 ^a^	5.50 ± 0.04 ^d^
40	2887 ± 115.97 ^c^	1708 ± 5.66 ^c^	1179 ± 110.31 ^c^	3013 ± 77.78 ^c^	1305 ± 72.12 ^d^	5.14 ± 0.09 ^b^

Note: Data with different letters in the same column were significantly different (*p* < 0.05) by Tukey’s test. Statistical analysis was only performed among the samples obtained from the same type of starch.

**Table 3:** Analysis of oxidative stability of deep-frying oil.

Sample	Deep-Fried Times/h	Peroxide Value/(meq/kg)	*p*-Anisidine Value	TOTOX
Maize starch				
	0	3.63 ± 0.54 ^a^	1.20 ± 0.02 ^a^	8.46 ± 1.06 ^a^
	8	4.79 ± 0.06 ^b^	63.14 ± 8.57 ^b^	72.73 ± 8.70 ^b^
	16	5.76 ± 0.44 ^cd^	73.14 ± 6.34 ^b^	84.66 ± 7.22 ^b^
	24	6.62 ± 0.36 ^d^	70.52 ± 1.99 ^b^	83.75 ± 2.72 ^b^
	32	5.15 ± 0.45 ^bc^	67.75 ± 0.86 ^b^	78.04 ± 1.77 ^b^
	40	4.64 ± 0.15 ^b^	66.25 ± 1.31 ^b^	75.52 ± 1.62 ^b^
Potato starch				
	0	3.63 ± 0.54 ^a^	1.20 ± 0.02 ^a^	8.46 ± 1.06 ^a^
	8	3.64 ± 0.15 ^a^	76.34 ± 3.48 ^b^	83.62 ± 3.19 ^b^
	16	4.22 ± 0.02 ^ab^	199.62 ± 5.32 ^c^	208.06 ± 5.36 ^c^
	24	4.54 ± 0.09 ^bc^	233.57 ± 5.05 ^e^	242.66 ± 4.87 ^e^
	32	5.09 ± 0.36 ^cd^	213.61 ± 5.11 ^d^	223.80 ± 4.39 ^d^
	40	5.48 ± 0.20 ^d^	71.77 ± 0.33 ^b^	82.73 ± 0.72 ^b^
High-amylose maize starch				
	0	3.63 ± 0.54 ^a^	1.20 ± 0.02 ^a^	8.46 ± 1.06 ^a^
	8	12.69 ± 12.69 ^c^	187.87 ± 0.26 ^b^	213.25 ± 1.79 ^b^
	16	15.35 ± 2.13 ^d^	298.97 ± 10.06 ^c^	329.68 ± 14.32 ^c^
	24	10.04 ± 0.76 ^b^	352.83 ± 5.42 ^d^	372.91 ± 3.89 ^d^
	32	8.19 ± 0.01 ^b^	331.74 ± 28.08 ^d^	348.12 ± 28.09 ^cd^
	40	7.55 ± 0.76 ^b^	327.38 ± 2.01 ^cd^	342.48 ± 0.49 ^cd^
Wheat starch				
	0	3.63 ± 0.54 ^a^	1.20 ± 0.02 ^a^	8.46 ± 1.06 ^a^
	8	7.98 ± 1.40 ^b^	232.17 ± 15.79 ^c^	248.12 ± 12.89 ^c^
	16	15.12 ± 0.85 ^d^	152.62 ± 5.05 ^b^	182.86 ± 3.35 ^b^
	24	10.60 ± 0.19 ^c^	256.08 ± 0.90 ^d^	277.28 ± 1.29 ^d^
	32	10.19 ± 1.70 ^bc^	308.71 ± 6.16 ^e^	329.09 ± 2.76 ^e^
	40	11.30 ± 0.41 ^c^	257.96 ± 6.64 ^d^	280.57 ± 5.82 ^d^

Note: Data with different letters in the same column were significantly different (*p* < 0.05) by Tukey’s test. Statistical analysis was only performed among the samples obtained from the same type of starch.

## Data Availability

Data is contained within the article or Supplementary Material.

## Supplementary Materials

The following supporting information can be downloaded at: https://www.mdpi.com/article/10.3390/foods11193083/s1, Figure S1: The amylose content of native maize starch, potato starch, high amylose maize starch, and wheat starch (0 h), as well as those over deep-frying process (8 h, 16 h, 24 h, 32 h, 40 h). (a) Maize starch; (b) Potato starch; (c) High amylose maize starch; (d) Wheat starch; Table S1: Moisture and lipid content of native starch samples.
