# Supplementary material for: Formation of Starch–Lipid Complexes during the Deep-Frying Process and Its Effects on Lipid Oxidation
Source: Foods. 2022 Oct 5;11(19):3083. doi: 10.3390/foods11193083 (PMC9562666; doi:10.3390/foods11193083)
Supplement: Supplementary file 1 [file foods-11-03083-s001.zip › foods-1913653-supplementary.pdf]

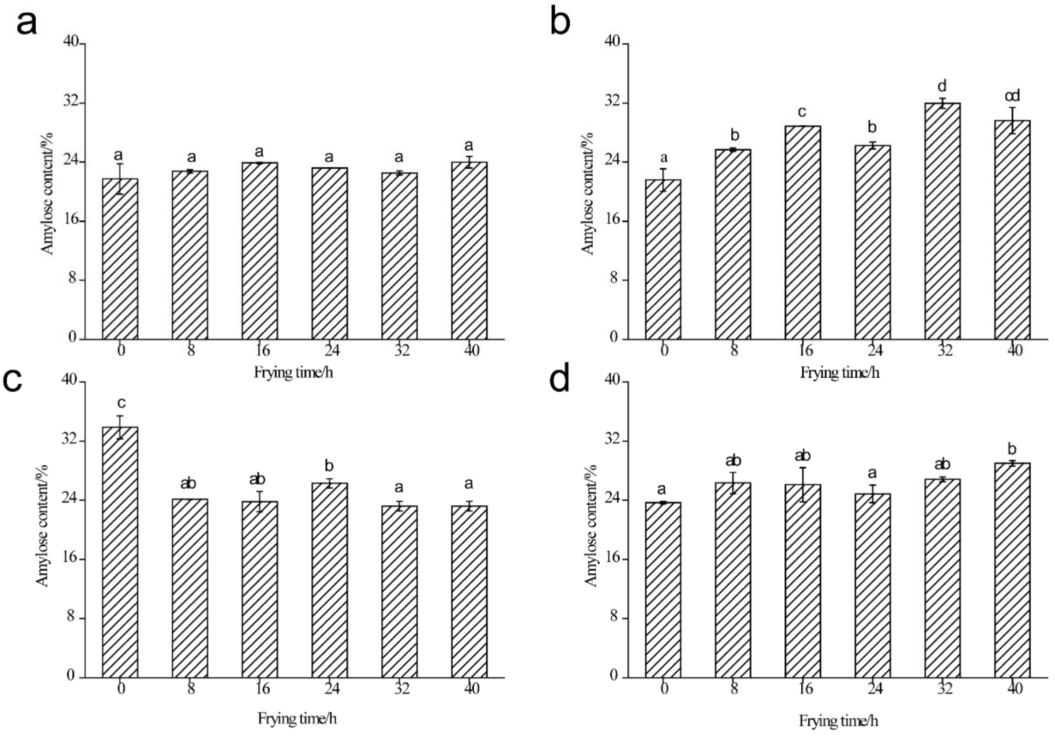

Figure S1. The amylose content of native maize starch, potato starch, high amylose maize starch, and wheat starch (0 h), as well as those over deep-frying process (8 h, 16 h, 24 h, 32 h, 40 h). (a) Maize starch; (b) Potato starch; (c) High amylose maize starch; (d) Wheat starch.

Table S1. Moisture and lipid content of native starch samples

Sample	Moisture content/%	lipid content/%
Maize starch	9.97 ± 0.31^{b}	5.22 ± 1.19^{a}
Potato starch	12.88 ± 0.02^{d}	5.90 ± 0.51^{a}
High-amylose maize starch	11.29 ± 0.04^{c}	10.84 ± 1.13^{b}
Wheat starch	9.28 ± 0.27^{a}	12.48 ± 0.14^{b}

Note: Data with different letters in the same column were significantly different ($p < 0.05$) by Tukey's test.
